# 25-hydroxyvitamin D levels in children of different ages and with varying degrees of *Helicobacter pylori* infection and immunological features

**DOI:** 10.3389/fped.2023.1157777

**Published:** 2023-04-17

**Authors:** Peng-fei Ma, Qun Dai, Jing Chu, Lin Zhuo, Yi Chen, Rong Cheng, Cheng Wu, Li-Ping Yuan

**Affiliations:** ^1^ Department of Pediatrics, The First Affiliated Hospital of Anhui Medical University, Hefei, China; ^2^ Department of Gastroenterology, Children's Hospital of Fudan University at Anhui (Anhui Provincial Children's Hospital), Hefei, China; ^3^ Department of Pathology, The Third People's Hospital of Hefei, Anhui Medical University Hefei Third Clinical College, Hefei, China; ^4^ Department of Pathology, Children's Hospital of Fudan University at Anhui (Anhui Provincial Children's Hospital), Hefei, China

**Keywords:** *helicobacter pylori* infection, 25(OH)D, lymphocyte subsets, inflammation, children

## Abstract

**Background:**

*Helicobacter pylori* (HP) is a major cause of upper digestive tract diseases. However, the relationship between HP infection and 25-hydroxyvitamin D [25(OH)D] levels in children has not been fully elucidated. This study investigated the levels of 25(OH)D in children of different ages and with varying degrees of HP infection and immunological features as well as the correlations between 25(OH)D levels in children infected with HP and their ages and degrees of infection.

**Materials and methods:**

Ninety-four children who underwent upper digestive endoscopy were divided into an HP-positive group without peptic ulcers (Group A), an HP-positive group with peptic ulcers (Group B) and an HP-negative control group (Group C). The serum levels of 25(OH)D and immunoglobulin and the percentages of lymphocyte subsets were determined. HP colonization, the degree of inflammation, and the degree of activity were further evaluated by HE staining and immunohistochemical staining in gastric mucosal biopsy.

**Results:**

The 25(OH)D level of the HP-positive groups (50.93 ± 16.51 nmol/L) was significantly lower than that of the HP-negative group (62.89 ± 19.18 nmol/L). The 25(OH)D level of Group B (47.79 ± 14.79 nmol/L) was lower than that of Group A (51.53 ± 17.05 nmol/L) and was significantly lower than that of Group C (62.89 ± 19.18 nmol/L). The 25(OH)D level decreased with increasing age, and there was a significant difference between Group C subjects who were ≤5 years old and those who were aged 6–9 years and ≥10 years. The 25(OH)D level was negatively correlated with HP colonization (*r* = −0.411, *P* < 0.01) and the degree of inflammation (*r* = −0.456, *P* < 0.01). The percentages of lymphocyte subsets and immunoglobulin levels among Groups A, B and C were not significantly different.

**Conclusions:**

The 25(OH)D level was negatively correlated with HP colonization and the degree of inflammation. As the age of the children increased, the level of 25(OH)D decreased, and the susceptibility to HP infection increased.

## 1. Introduction

*Helicobacter pylori* (HP) is a major cause of upper digestive tract diseases, including chronic and atrophic gastritis, peptic ulcer disease, mucosa-associated lymphoid tissue lymphoma, and gastric adenocarcinoma (1, 2). HP was first discovered in the stomachs of patients with gastritis and ulcers by Marshall and Warren in 1982. Approximately one-third of children worldwide are infected with HP, and the prevalence varies among different regions of the world (2). In China, there are also considerable regional disparities in the incidence of HP infection in children, with infection rates ranging from 30% to 60% (3, 4). HP infection in children can affect their growth and development, nutritional metabolism, and autoimmune systems (5) and is related to 25-hydroxyvitamin D [25(OH)D] deficiency (6), which could be a risk factor for HP eradication failure (7). However, there are few studies on the correlation between the levels of 25(OH)D in children with different degrees of HP infection and immunity.

25(OH)D is a steroid hormone derived from vitamin D and is the main indicator of vitamin D levels in the body. In recent years, this hormone has been shown to play a very important role in tumor prevention, immune system regulation, promotion of some types of cell differentiation, and anti-cell proliferation (8). In this study, the relationships between 25(OH)D levels in children and their ages, degrees of HP infection and levels of immunoglobulin and lymphocytes were investigated to provide novel insights into the prevention and treatment of HP infection in children.

## 2. Materials and methods

### 2.1. Study design and patients

Children with alarm signs who underwent upper digestive endoscopy in the Gastroenterology Department of Anhui Provincial Children's Hospital from March 2021 to September 2022 were identified. Alarm signs included persistent upper right or lower right quadrant pain, dysphagia, odynophagia, persistent vomiting, and gastrointestinal blood loss. These children underwent a ^13^C breath test and gastric mucosal tissue biopsy, and serum 25(OH)D and immunoglobulin levels and lymphocyte subset percentages were measured. In addition, these children did not take antibiotics, probiotics, antacids, bismuth, or traditional Chinese medicine with antibacterial effects in the previous 1 month. The exclusion criteria were a possible combination of other diseases, such as Henoch purpura, nephritis, inflammatory bowel disease, and connective tissue disease. Children meeting the diagnostic criteria of HP infection (9) were divided into an HP-positive group without peptic ulcers (Group A) and an HP-positive group with peptic ulcers (Group B) according to gastroscopic diagnosis results. Moreover, the children who were confirmed to have superficial gastritis by upper digestive endoscopy and those who had negative ^13^C and RUT tests and no HP detected on histopathology of the gastric mucosa were chosen as the control group (Group C).

Upper digestive endoscopy was performed by experienced endoscopy experts, and the Olympus GIF-H290 (8.9 mm) and Olympus GIF-XP290 (5.8 mm) (Olympus, Japan) upper digestive endoscopes were used. The endoscope used was selected according to the age of the child. All the children underwent gastric mucosal tissue biopsy and a rapid urease test during the examination, and then gastric mucosal tissue biopsy specimens were fixed with tissue fixation solution. Based on diagnostic criteria, superficial gastritis, HP-related gastritis, and peptic ulcers were identified (10). This study was approved by the Ethics Committee of Anhui Provincial Children's Hospital (EYLL-2017-012). Written informed consent was obtained from the guardian of each subject before endoscopy.

### 2.2. Determination of serum 25(OH)D and immunoglobulin levels and lymphocyte subsets

Determination of 25(OH)D: Three milliliters of venous blood was obtained from each child and centrifuged at 3,000 r/min for 10 min. The serum 25(OH)D level was detected by chemiluminescence immunoassay.

Immunoglobulin assay: IgG, IgA, and IgM levels in serum were determined with a fully automated biochemical analyzer.

Detection of lymphocyte subset percentages: Three milliliters of venous blood was obtained from each child, centrifuged at 1,600 r/min for 8 min, and analyzed by flow cytometry.

### 2.3. Evaluation of HP colonization, inflammation and activity in gastric mucosal tissues

The gastric mucosal biopsy tissues were embedded in paraffin and sectioned, and the sections were stained with hematoxylin-eosin (HE) for the evaluation of tissue inflammation. Another section was taken for immunohistochemical staining to evaluate HP colonization. The degree of inflammation was evaluated according to the degree of infiltration of lymphocytes and plasma cells into the glandular epithelium and the inherent membrane. The degree of activity was evaluated according to the degree of infiltration of neutrophils into the glandular epithelium and the inherent membrane. HP colonization was evaluated according to the amount of HP in the mucous layer of the gastric mucosa. The degree of inflammation, degree of activity, and HP colonization were divided into 4 grades, as described previously (11, 12). A score of 0–3 was assigned according to the grade (none = 0; mild = 1; medium = 2; heavy = 3). Each section was evaluated by two senior pathologists, and the evaluation criteria were based on the evaluation criteria and diagnostic criteria of gastric mucosal inflammation in HP-infected patients (11).

### 2.4. Statistical analysis

Statistical Package for Social Sciences Version 27.0 (SPSS, IBM) was used to analyze the data, and GraphPad Prism 9.0 software was used to generate figures. The approximate normal distribution measurement data are expressed as the mean ± standard deviation (*χ* ± SD). The differences between two groups and among multiple groups were determined by unpaired *t* test and ANOVA, respectively. Spearman rank correlation analysis was used to analyze the correlation between measurement data and grade data. Statistical significance was defined as *P* < 0.05.

## 3. Results

### 3.1. Patients

A total of 94 children were enrolled, of which 53 were males and 41 were females. The average age was 7.89 ± 3.18 years (2.6–15 years). There were 39 patients in Group A, and the average age of this group was 8.08 ± 3.22 years. There were 8 patients in Group B, and the average age of this group was 8.50 ± 3.02 years. There were 47 patients in Group C, and the average age of this group was 7.64 ± 3.21 years. There were no significant differences in age among the three groups.

### 3.2. Serum 25(OH)D levels, lymphocyte subset percentages and immunoglobulin levels in children of various ages with varying degrees of HP infection

**Figure 1:**
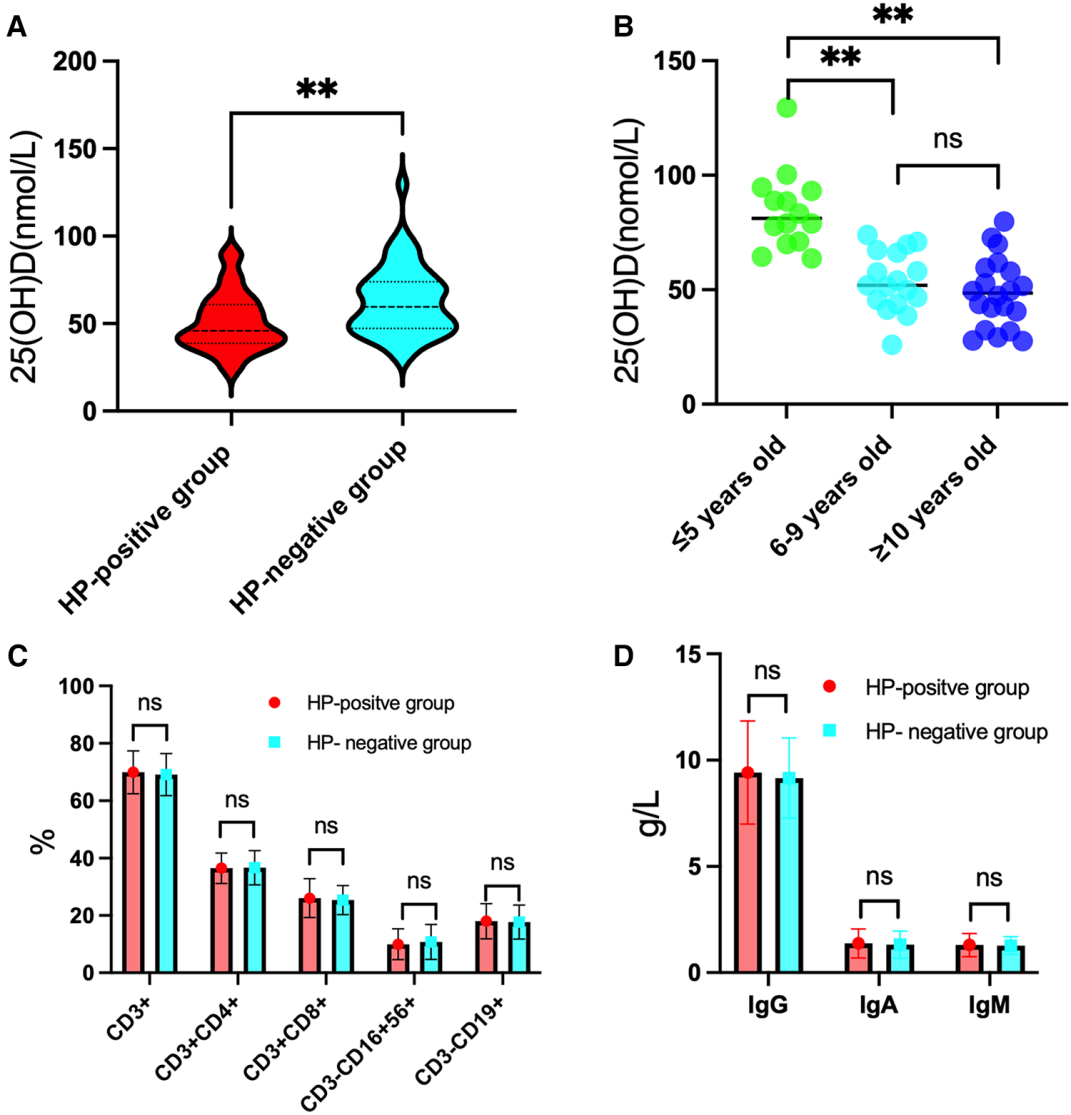
Results for 25(OH)D levels, lymphocyte subset percentages and immunoglobulin levels in the HP-positive groups in comparison to the HP-negative group. (**A**) The level of 25(OH)D was significantly lower in the HP-positive groups than in the HP-negative group. (**B**) In the HP-negative control group, the level of 25(OH)D in the ≤5-year-old group was significantly higher than those in the 6–9-year-old group and the ≥10-year-old group. (**C**) Comparison of the percentages of T and B lymphocyte subsets between the HP-positive groups and the HP-negative control group. (**D**) Comparison of the immunoglobulin levels between the HP-positive groups and the HP-negative control group. **P < 0.01, “ns” means not statistically significant.

The 25(OH)D level in the HP-positive groups (50.93 ± 16.51 nmol/L) was significantly lower than that in the HP-negative control group (62.89 ± 19.18 nmol/L) (*P* < 0.01), as shown in Figure 1A. Moreover, the serum 25(OH)D level of HP-infected children with different degrees of infection and ages was determined. The 25(OH)D level of HP-infected children with ulcers (Group B) was lower than that of HP-infected children without ulcers (Group A) and significantly lower than that of the HP-negative control group (Group C). These differences were significant (*P* < 0.01), as shown in Table 1. The serum 25(OH)D level of Group A was significantly lower than that of Group C in patients ≤5 years old and ≥10 years old (all *P* < 0.05). In addition, the serum 25(OH)D levels in Group A and Group C decreased with increasing age. The serum 25(OH)D levels in Group C were significantly different among the ≤5-year-old group, the 6–9-year-old group, and the ≥10-year-old group (all *P* < 0.01), as shown in Figure 1B.

**Table 1:** Serum 25(OH)D levels of children in the three groups according to different ages within each group (*x* ± SD).

	*n*	Group A	*n*	Group B	*n*	Group C
≤5 years old	10	62.77 ± 16.89**	/	/	14	84.51 ± 17.16
6–9 years old	15	52.34 ± 18.58	/	/	16	55.43 ± 11.27
≥10 years old	14	41.84 ± 8.52*	/	/	17	52.10 ± 13.51
Total	39	51.53 ± 17.05**	8	47.79 ± 14.79**	47	62.89 ± 19.18

* *P* < 0.05.

** *P* < 0.01, compared to Group C; “*n*” is the number of examples; “/” indicates that the number of cases is too few to be grouped by age.

There were no statistically significant differences in the percentages of T and B lymphocyte subsets and immunoglobulin levels in peripheral blood between the HP-positive and HP-negative groups (Figures 1C,D) or among Groups A, B, and C (Table 2).

**Table 2:** Percentages of T and B lymphocyte subsets and immunoglobulin levels in the peripheral blood of each group (*x* ± SD).

	Group A	Group B	Group C	*P* values
CD3 + (%)	70.41 ± 7.49	67.56 ± 7.14	69.11 ± 7.35	0.540
CD3 + CD4 + (%)	36.54 ± 5.55	36.38 ± 4.11	36.67 ± 5.99	0.989
CD3 + CD8 + (%)	26.40 ± 6.51	24.36 ± 8.30	25.35 ± 6.05	0.600
CD3 + CD4+/CD3 + CD8+	1.48 ± 0.48	1.59 ± 0.39	1.51 ± 0.41	0.774
CD3-CD16 + 56 + (%)	9.85 ± 5.42	10.63 ± 5.35	10.77 ± 6.08	0.767
CD3-CD19 + (%)	17.78 ± 6.06	19.19 ± 6.73	17.71 ± 5.88	0.813
IgG (g/L)	9.67 ± 2.42	8.20 ± 2.22	9.16 ± 1.88	0.186
IgA (g/L)	1.40 ± 0.71	1.28 ± 0.56	1.32 ± 0.64	0.823
IgM (g/L)	1.34 ± 0.54	1.08 ± 0.56	1.27 ± 0.42	0.345

### 3.3. Evaluation of HP colonization, degree of inflammation, and degree of activity in gastric mucosal tissue

Gastric mucosal biopsies from 39 children in Group A, 8 children in Group B, and 10 randomly selected children in Group C were evaluated.

**Figure 2:**
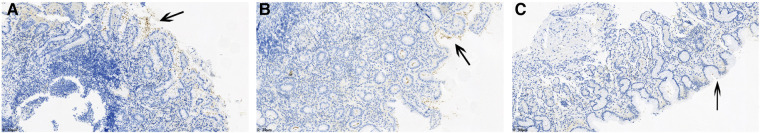
Immunohistochemical staining results showed the degree of HP colonization in the gastric mucosa. (**A**) A concentrated amount of HP bacilli (black arrow) was found on the surface of the gastric mucosa, the gastric pits, and the epithelial surface of the glandular ducts (severe). (**B**) A medium amount of HP bacilli was found on the surface of the gastric mucosa, the gastric pits, and the epithelial surface of the glandular ducts (moderate). (**C**) Small amounts of HP bacilli were found on the surface of the gastric mucosa, the gastric pits and the epithelial surface of the glandular ducts (mild).

The total detection rate of the HP-positive groups was 93.6% (44/47). Immunohistochemical staining showed that HP was rod-shaped on the surface of the mucous layer, surface epithelium, concave epithelium, and glandular duct epithelium of the gastric mucosa, as shown in Figures 2A–C. The grading of HP colonization was analyzed as previously described (12), and the HP colonization in Group A was as follows: 25.6% (10/39) of patients had severe colonization, 12.8% (5/39) had moderate, and 53.8% (21/39) had mild. HP colonization was severe in 100% (8/8) of patients in Group B. No HP was found in Group C. The scores of HP colonization were significantly different between the HP-positive and HP-negative groups (Figure 4A). The HP colonization scores in Group B were significantly higher than those in Group A (3.00 ± 0.00 vs. 1.56 ± 0.97 points, *P* < 0.001) (Table 3).

**Figure 3:**
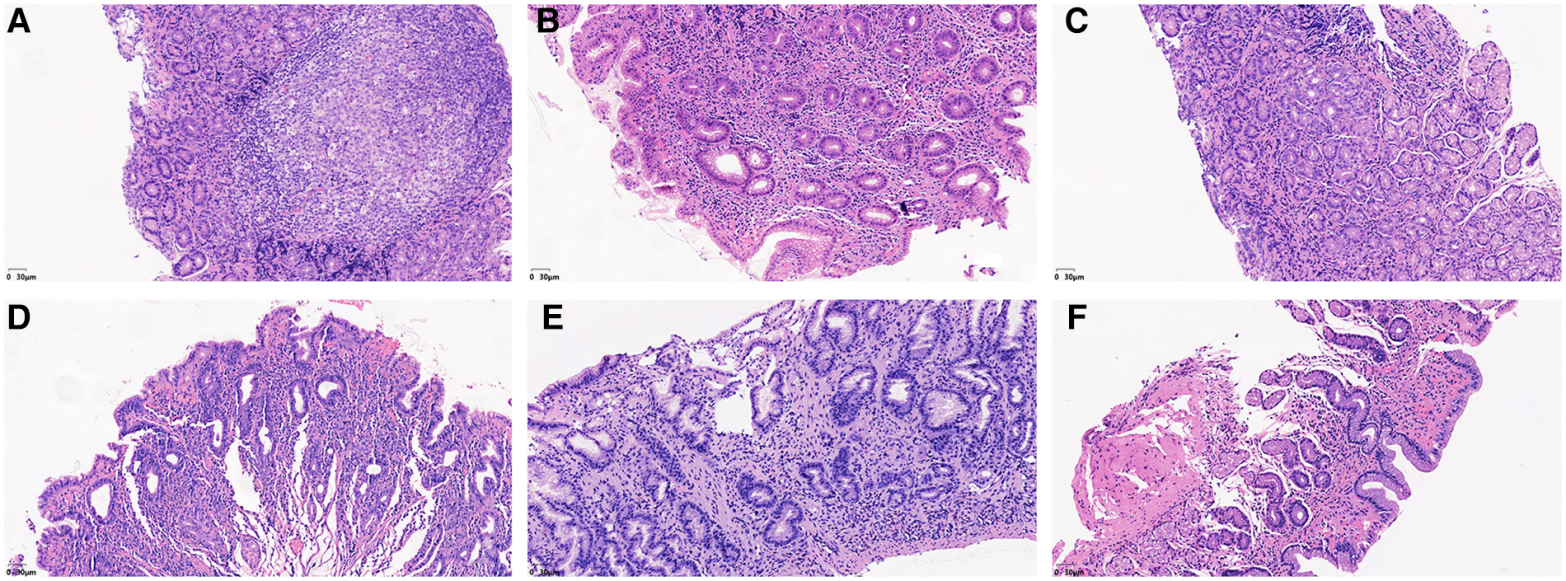
He staining results showed the degree of lymphocyte cells, plasma cells and neutrophils in the gastric mucosa. (**A**) Lymphocytes and plasma cells accumulated in the epithelial cells and lamina propria of the gastric mucosa (severe). (**B**) Small amounts of lymphocytes and plasma cells were found in the gastric mucosal epithelium, and considerable amounts were found in the lamina propria (moderate). (**C**) Small amounts of lymphocytes and plasma cells were found in the epithelium and lamina propria of the gastric mucosa (mild). (**D**) Neutrophils accumulated in the epithelial cell layer and lamina propria of the gastric mucosa (severe). (**E**) Small amounts of neutrophils were found in the gastric mucosal epithelial cell layer, while considerable amounts were found in the lamina propria (moderate). (**F**) Small amounts of neutrophils were found in the epithelial cell layer and lamina propria of the gastric mucosa (mild).

**Table 3:** Scores of HP colonization, degree of inflammation and degree of activity in each group (*x* ± SD).

Group	The amount of HP colonization	The degree of inflammation	The degree of inflammatory activity
Group A	1.56 ± 0.97^a^	1.77 ± 0.87^a^	1.15 ± 0.67^a^
Group B	3.00 ± 0.00^b^	2.63 ± 0.52^b^	2.88 ± 0.35^b^
Group C	0.00 ± 0.00	0.70 ± 0.67	0.40 ± 0.52

^a^ *P* < 0.05 compared with Group C.

^b^ *P* < 0.05 compared to Group A.

**Figure 4:**
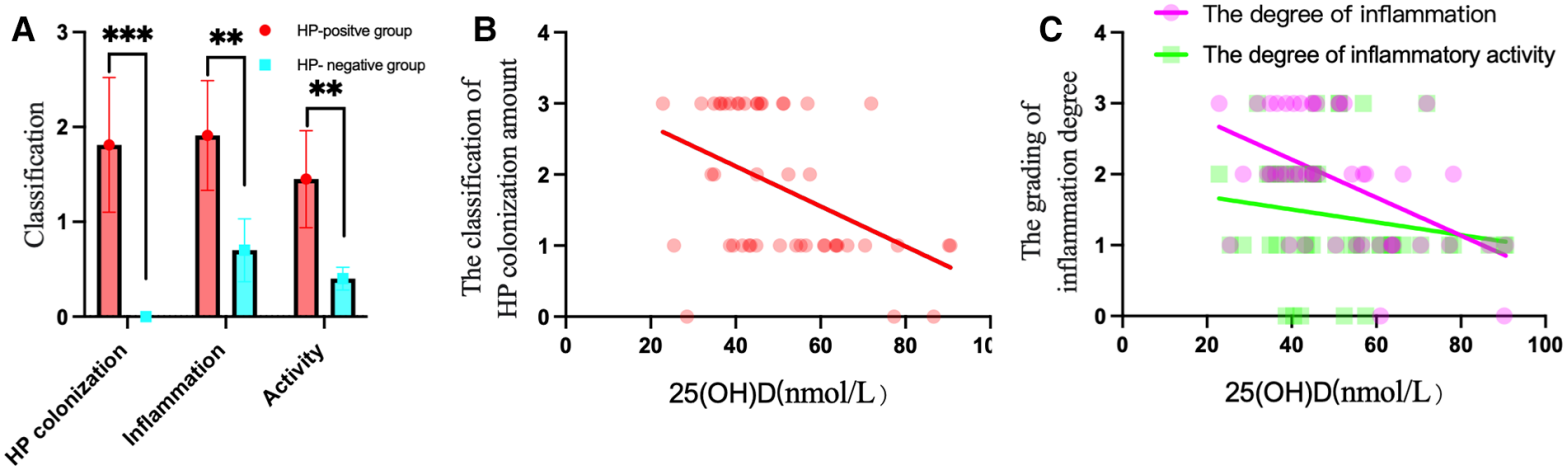
HP colonization, inflammation and activity in the HP-positive groups were compared to those in the HP-negative group, and correlations with 25(OH)D levels were found in the HP-positive groups. (**A**) The scores of HP colonization, inflammation, and activity in the HP-positive groups were significantly higher than those in the HP-negative group. **P < 0.01, ***P < 0.001. (**B**) 25(OH)D was negatively correlated with the amount of HP colonization of the gastric mucosa. (**C**) 25(OH)D was negatively correlated with the degree of inflammation of the gastric mucosa.

No lymphocytes or plasma cells were found in the normal gastric mucosal epithelial cell layer and lamina propria. After HP infection, the epithelial layer and lamina propria showed varying degrees of lymphocyte and plasma cell infiltration. In particular, lymphocyte and plasma cell aggregation was observed in the HP-positive group with ulcers, as shown in Figures 3A–C. The grading of inflammation degree was analyzed as previously described (11), and the degree of inflammation in Group A was as follows: 23.1% (9/39) of patients had severe inflammation, 35.9% (14/39) had moderate, and 35.9% (14/39) had mild. In Group B, severe inflammation was observed in 62.5% (5/8) of patients, and moderate inflammation in 37.5% (3/8). The HP inflammation scores were significantly different between the HP-positive and HP-negative groups (Figure 4A). The inflammation score in Group B was significantly higher than that in Group A (2.63 ± 0.52 points vs. 1.77 ± 0.87 points, *P* < 0.05) (Table 3).

No neutrophils were found in the normal gastric mucosal epithelial cell layer and lamina propria. After HP infection, the epithelial layer and lamina propria showed varying degrees of neutrophil infiltration. In particular, neutrophil aggregation was observed in the HP-infected ulcer group, as shown in Figures 3D–F. The grading of activity degree was analyzed as previously described (11). In Group A, severe activity was observed in 2.6% (1/39) of patients, moderate activity in 23.1% (9/39), and mild activity in 61.5% (24/39). In Group B, severe activity was observed in 87.5% (7/8) of patients and moderate activity in 12.5% (1/8). The HP activity degree scores were significantly different between the HP-positive and HP-negative groups (Figure 4A). The activity degree score in Group B was significantly higher than that in Group A (2.88 ± 0.35 points vs. 1.15 ± 0.67 points, *P* < 0.001) (Table 3).

### 3.4. Relationship between serum 25(OH)D levels and HP colonization and inflammation in gastric mucosa in children with HP

The 25(OH)D level was negatively correlated with HP colonization (*r* = −0.411, *P* < 0.01) and the degree of inflammation (*r* = −0.456, *P* < 0.01), as shown in Figures 4B,C, which suggested that the higher the HP colonization is, the higher the degree of mucosal inflammation and the lower the serum 25(OH)D level.

## 4. Discussion

It has been reported that low gastric acid levels caused by HP-related chronic gastritis can lead to reduced absorption of iron and vitamin B12 as well as other micronutrients and vitamin D (13). However, the relationship between 25(OH)D levels and different ages and degrees of HP infection in children has not been reported thus far. In our study, we found that HP colonization in the mucosa was positively correlated with the degree of gastric mucosal inflammation and activity. However, serum 25(OH)D levels were negatively correlated with both HP colonization and the degree of inflammation in the gastric mucosa. These findings suggested that vitamin D absorption was impacted by HP infection and that the impact increased as the degree of infection escalated. Our study unexpectedly discovered that serum 25(OH)D levels in five HP-infected children increased following eradication therapy compared to the prior period, despite these children neither taking vitamin D supplements nor making any changes to their lifestyle (Supplementary Table S1). This finding reinforces the view that HP infection significantly affects vitamin D absorption.

Vitamin D deficiency may increase the risk of HP infection (14). Zhou (15) found that the CagA content of mice with vitamin D receptor (VDR) knockout was significantly higher than that of wild-type mice, indicating that VDR knockout increased the susceptibility of mice to HP infection. El Shahawy (16) demonstrated that the success rate of eradication in infected patients with vitamin D deficiency is low and that serum vitamin D deficiency may be an independent risk factor for treatment failure in HP infection (17). Long-term oral vitamin D supplementation has been shown to reduce the risk of HP infection (18). Nevertheless, epidemiological research on vitamin D levels in Chinese children is lacking. In China, the vast majority of children take vitamin D supplements until they are 3 years old. Very few children are not supplemented with vitamin D after birth. In 2022, the Subspecialty Group of Children Health and Editorial Board issued guidelines recommending oral vitamin D supplementation (400 U/d) in China until adolescence (19), which will be generalized nationwide. Our study found that the serum 25(OH)D level of children in the HP-negative control group decreased with increasing age, especially in children older than 6 years. This was mainly due to parental neglect regarding vitamin D supplementation and a decrease in outdoor activities due to lifestyle changes. Ren (3) and Yuan (20) studied the prevalence of HP infection in children in China and worldwide and found that the HP infection rate increased with increasing age. These findings show that the risk of HP infection increases with decreasing 25(OH)D levels. Vitamin D has been shown to inhibit and clear HP through a variety of pathways. 1,25(OH)2D3 supports the integrity of the intestinal barrier by enhancing the action of antibacterial proteins, such as cathelicidin and *β*-defensin, in macrophages and monocytes. Thus, it regulates intestinal microbiome composition and protects the normal bacterial community. In addition, it enhances the differentiation of monocytes into macrophages and enhances the motility and phagocytosis ability of macrophages. Calcitriol downregulates IFN-*γ* and proinflammatory cytokine production by increasing the expression of anti-inflammatory cytokines (21). The breakdown product of vitamin D3 (VDP1) leads to the collapse of the membrane structure of HP and plays an antibacterial role (22). Hu et al. (23) proposed that vitamin D3 can remove HP by reactivating lysosome acidification and degradation functions by activating the PDIA3/STAT3-MCOLN3-Ca2+ axis. In addition, Tsai et al. proposed that the frequency of upper digestive tract bleeding caused by peptic ulcers has seasonal changes, with a higher incidence in winter and spring and a lower incidence in summer (24). Additionally, approximately two-thirds of peptic ulcers in children are caused by HP infection (25), which further supports the above views.

Infection with HP in adults increases the number of CD4+ T cells and induces the activation of CD4+ and CD8+ T cells (26). T helper type 1 (Th1) cells specifically recognize the HP antigen. Th1 cells promote the expression of the cytokine IL-1β, which increases the risk of hypogastric acid, gastric atrophy, and gastric adenocarcinoma. Regulatory T cells (Tregs) and the Th2-initiated immune system are protective responses to specific HP antigens that contribute to the alleviation of host tissue damage and HP colonization (27, 28). However, some studies (28) have found no significant differences in the percentages of T and B lymphocyte subsets in the peripheral blood of children with different degrees of HP infection, which is consistent with the results of our study. Furthermore, there was no significant difference in immunoglobulin levels among children with different degrees of HP infection. This finding may be explained as follows: (1) the local and systemic proinflammatory responses to HP infection in children are not as strong as those in adults, and (2) the downregulation of the HP-induced immune response may also be related to the immaturity of the immune response (29).

This study had some limitations. The sample size was small, and the critical value of the serum 25(OH)D level for HP susceptibility was not determined. Therefore, larger prospective clinical trials with increased consideration of pretreatment vitamin D levels are needed to further evaluate the relationship between vitamin D status and HP infection.

In summary, this study showed that the degree of inflammation and activity in the gastric mucosa were positively correlated with HP colonization in the gastric mucosa and that the level of 25(OH)D was negatively correlated with HP colonization and the degree of inflammation. As the age of children increased, the level of 25(OH)D decreased, and the susceptibility to HP infection increased.

## Data Availability

The original contributions presented in the study are included in the article/**Supplementary Material**, further inquiries can be directed to the corresponding author.
