# Supplementary material for: 25-hydroxyvitamin D levels in children of different ages and with varying degrees of Helicobacter pylori infection and immunological features
Source: Front Pediatr. 2023 Apr 17;11:1157777. doi: 10.3389/fped.2023.1157777 (PMC10149923; doi:10.3389/fped.2023.1157777)
Supplement: Supplementary file 1 [file Table1.docx]

**Supplemental table 1** Differences in 25(OH)D levels before and after

HP eradication treatment

| Patients | 25(OH)D levels before therapy(B) | 25(OH)D levels after therapy(A) | A-B |
| --- | --- | --- | --- |
| 1 | 38.7 | 47.2 | 8.7 |
| 2 | 46.04 | 54.98 | 8.94 |
| 3 | 31.9 | 42.11 | 10.21 |
| 4 | 56.89 | 63.25 | 6.36 |
| 5 | 44.98 | 52.87 | 7.89 |
| x ± SD | / | / | 8.42±1.42 |

Note: The unit of 25(OH)D level is nmol/L.
